# Fumarate Analogs Act as Allosteric Inhibitors of the Human Mitochondrial NAD(P)^+^-Dependent Malic Enzyme

**DOI:** 10.1371/journal.pone.0098385

**Published:** 2014-06-09

**Authors:** Ju-Yi Hsieh, Jyung-Hurng Liu, Pai-Chun Yang, Chi-Li Lin, Guang-Yaw Liu, Hui-Chih Hung

**Affiliations:** 1 Department of Life Sciences, National Chung Hsing University, Taichung, Taiwan; 2 Institute of Genomics and Bioinformatics, National Chung Hsing University, Taichung, Taiwan; 3 Institute of Medicine, Chung Shan Medical University, Taichung, Taiwan; 4 Institute of Microbiology & Immunology, Chung Shan Medical University, and Division of Allergy, Immunology, and Rheumatology, Chung Shan Medical University Hospital, Taichung, Taiwan; 5 Agricultural Biotechnology Center (ABC), National Chung Hsing University, Taichung, Taiwan; Centro de Biología Molecular Severo Ochoa (CSIC-UAM), Spain

## Abstract

Human mitochondrial NAD(P)^+^-dependent malic enzyme (m-NAD(P)-ME) is allosterically activated by the four-carbon *trans* dicarboxylic acid, fumarate. Previous studies have suggested that the dicarboxylic acid in a *trans* conformation around the carbon-carbon double bond is required for the allosteric activation of the enzyme. In this paper, the allosteric effects of fumarate analogs on m-NAD(P)-ME are investigated. Two fumarate-insensitive mutants, m-NAD(P)-ME_R67A/R91A and m-NAD(P)-ME_K57S/E59N/K73E/D102S, as well as c-NADP-ME, were used as the negative controls. Among these analogs, mesaconate, *trans*-aconitate, monomethyl fumarate and monoethyl fumarate were allosteric activators of the enzyme, while oxaloacetate, diethyl oxalacetate, and dimethyl fumarate were found to be allosteric inhibitors of human m-NAD(P)-ME. The IC_50_ value for diethyl oxalacetate was approximately 2.5 mM. This paper suggests that the allosteric inhibitors may impede the conformational change from open form to closed form and therefore inhibit m-NAD(P)-ME enzyme activity.

## Introduction

Malic enzymes (MEs) are a family of Mg^2+^ or Mn^2+^-dependent oxidative decarboxylases that catalyze L-malate to CO_2_ and pyruvate, with a concomitant reduction of NAD(P)^+^ to NAD(P)H [1]–[4]. Malic enzymes are abundant in all species from bacteria to humans. In mammals, there are three isoforms distributed within the cells according to their subcellular localization and cofactor specificity: the cytosolic NADP^+^-dependent malic enzyme (c-NADP-ME, ME1) [5], [6], the mitochondrial NAD(P)^+^-dependent malic enzyme (m-NAD(P)-ME, ME2) [2], [7], [8] and the mitochondrial NADP^+^-dependent malic enzyme (m-NADP-ME, ME3) [9]. c-NADP-ME is expressed in the liver and adipose tissues [1], [5] and generates the NADPH required for the biosynthesis of long-chain fatty acids and steroids [1], [5], [7], [9] m-NADP-ME is found in tissues with low division rates, such as heart, muscle and brain tissue, and it also generates the NADPH for fatty acid biosynthesis [1]. The m-NAD(P)-ME isoform can use either NAD^+^ or NADP^+^ as a cofactor in the catalytic reaction, and therefore, this enzyme generates NADH and NADPH in the mitochondria and may play dual roles in energy production and reductive biosynthesis [2], [10], [11]. Furthermore, m-NAD(P)-ME is exclusively regulated by the TCA cycle intermediate, fumarate, which acts as an allosteric activator of the enzyme. The enzyme is also inhibited by ATP, but the ATP binding sites differ from the fumarate binding sites on the enzyme [12]–[14].

There is growing evidence that the m-NAD(P)-ME is involved in tumor growth and transformation because it is overexpressed in tumors and is required for optimal NADPH production, glutaminolysis and lipid synthesis [7], [8], [11], [15]–[19]. Recently studies showed that p53 inversely regulates these metabolic pathways via m-NAD(P)-ME repression [20], [21]. Because allosteric regulation is unique to m-NAD(P)-ME and may subsequently have profound effects on cancer cell metabolism, designing or discovering allosteric inhibitors for this enzyme may be important for cancer therapy.

Various structures of human m-NAD(P)-ME, including an open form without substrate and metal ion and a closed form with a bound substrate analog and all cofactors and regulators, are available [12], [22]–[24]. In the structure of the enzyme, two regulatory sites can be found in proximity to the active site. One site is located at the dimer interface and is occupied by the allosteric activator, fumarate [12], [25]; the other site is located at the tetramer interface and is occupied by either an NAD or an ATP molecule. In the structure of the fumarate-bound enzyme, fumarate is ion-paired with Arg67 and Arg91. When these Arg residues are mutated, the fumarate activating effect is completely abolished [12]. Furthermore, earlier studies by our group demonstrated that some ionic amino acid residues that are not conserved among the different malic enzyme isoforms, including Lys57, Glu59, Lys73 and Asp102, have remarkable effects on fumarate-induced activation [12], [26]–[28]. We also examined the effect of structural analogs of the substrate malate and the allosteric activator fumarate on human m-NAD(P)-ME and, therefore, suggest that dicarboxylic acid in a *trans* conformation around the carbon-carbon double bond is required for the allosteric activation of the enzyme.

In this paper, the effects of fumarate analogs on m-NAD(P)-ME and c-NADP-ME were investigated. Among these analogs, diethyl oxaloacetate was found to be an allosteric inhibitor of human m-NAD(P)-ME.

## Results and Discussion

As previously mentioned, fumarate is the allosteric activator of human m-NAD(P)-ME [13]. Several residues have been shown to interact with fumarate directly or indirectly. The direct residues are Arg67 and Arg91, and the indirect residues are Lys57, Asp59, Lys73 and Asp102. Mutation of these residues causes the loss of the fumarate activating effect. Additionally, c-NADP-ME is unresponsive to fumarate activation. Here, the two fumarate-insensitive mutants, m-NAD(P)-ME_R67A/R91A and m-NAD(P)-ME_K57S/E59N/K73E/D102S, as well as c-NADP-ME, were used as the negative controls.

### Allosteric Activation of Human m-NAD(P)-ME by Fumarate and its Analogs

**Figure 1:**
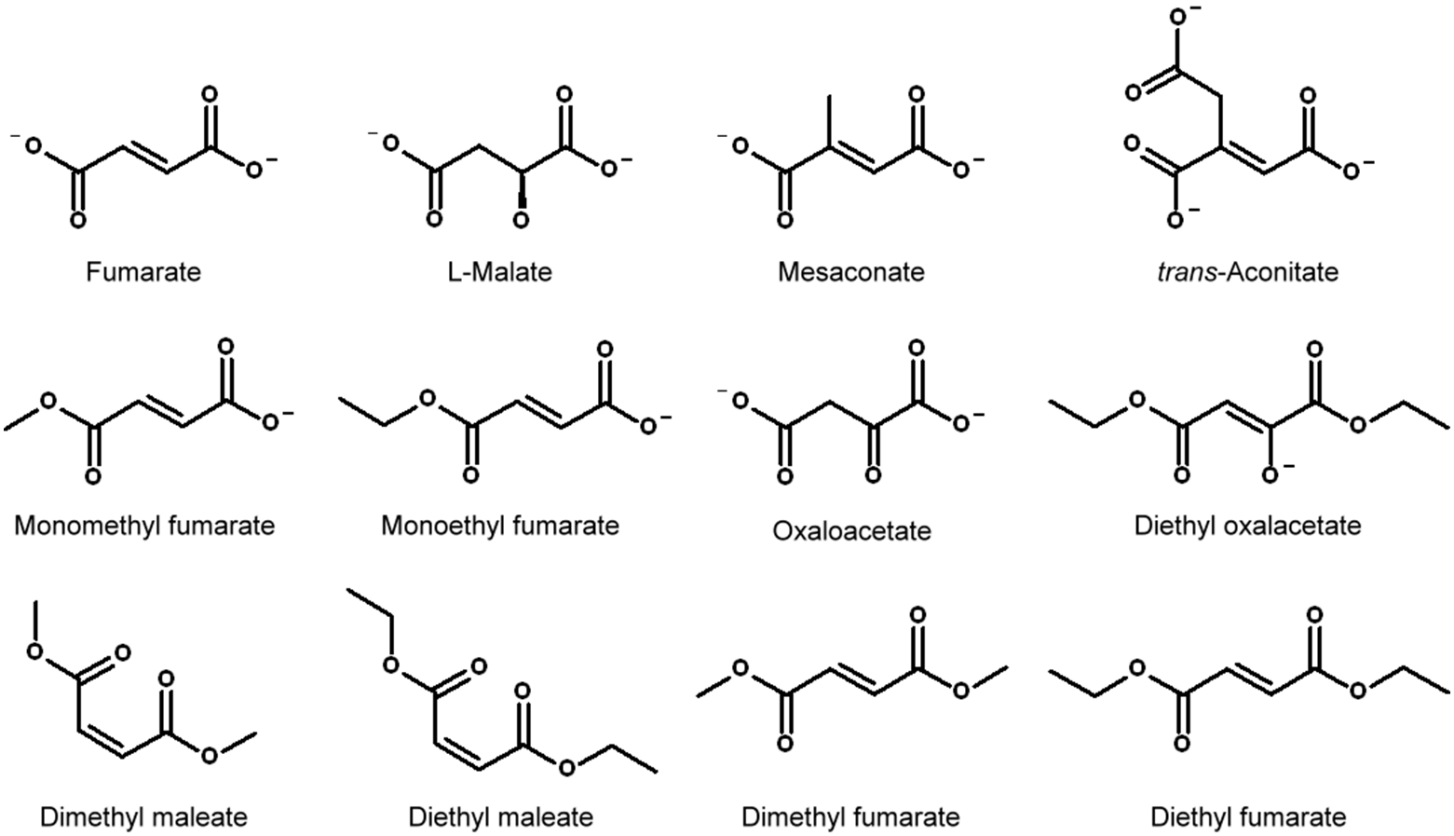
Chemical structures of fumarate and its analogs. These structures were generated using Accelrys Draw (Accelrys, USA).

**Figure 2:**
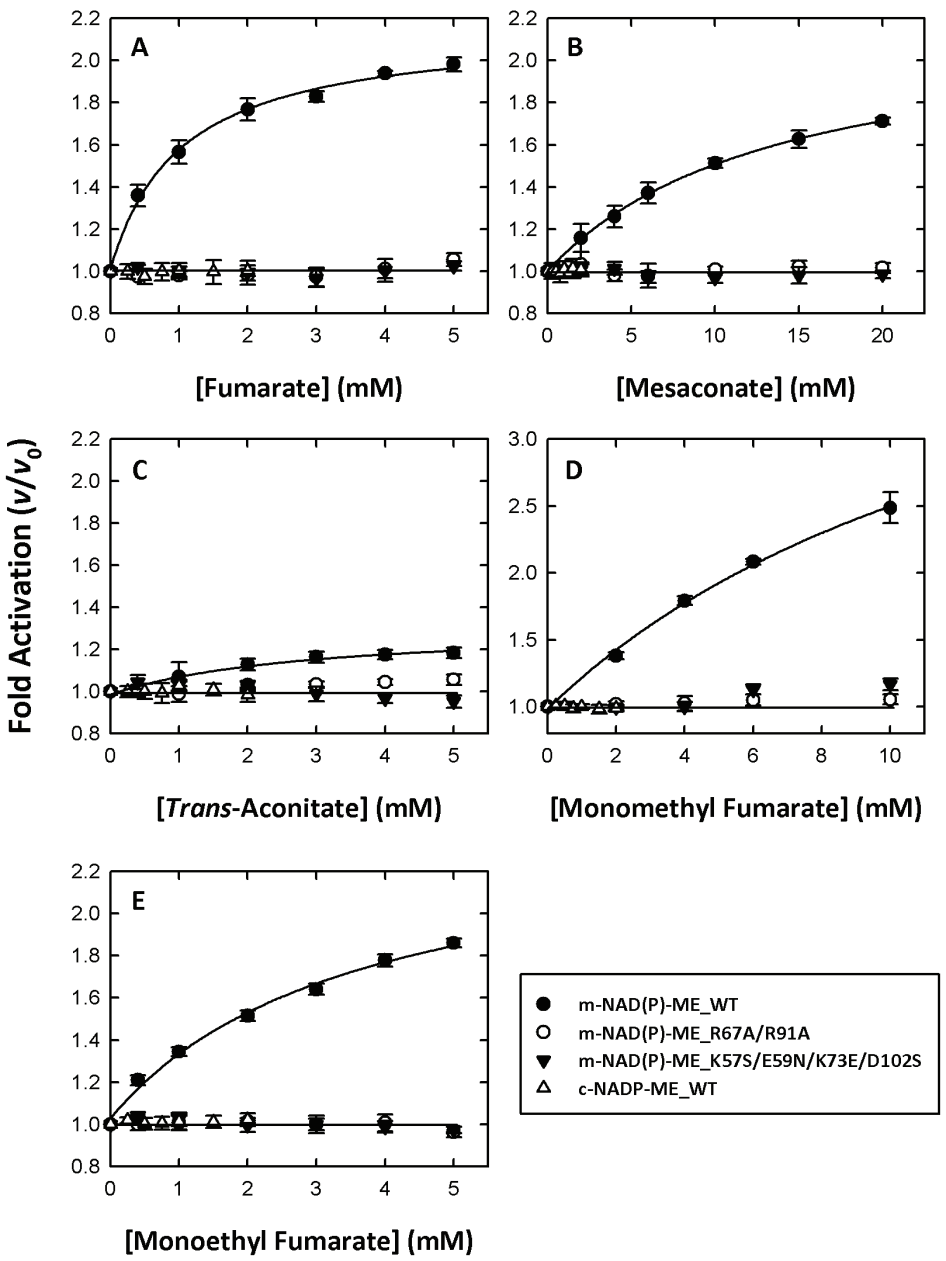
Allosteric activation of human m-NAD(P)-ME by fumarate and its analogs. (A) Fumarate; (B) Mesaconate; (C) *Trans*-aconitate; (D) Monomethyl fumarate; (E) Monoethyl fumarate. Closed circles, m-NAD(P)-ME; open circles, m-NAD(P)-ME_R67A/R91A; closed triangles, m-NAD(P)-ME_K57S/E59N/K73E/D102S; open triangles, c -NADP-ME. The specific activities of the m-NADP-(P)-ME WT, R67A/R91A, K57S/E59N/K73E/D102S and c-NADP-ME WT were approximately 0.1, 0.005, 0.02 and 0.08 µmol/min, respectively, and the final enzyme concentration in an individual assay was 20, 400, 100, and 25 ng/µl, respectively. The v and v_0_ represented the enzyme activity in the presence and absence of fumarate analogs, respectively.

Figure 1 shows the chemical structures of fumarate and its analogs and Figure 2 shows the activating effects of fumarate and its analogs on m-NAD(P)-ME. Fumarate can activate the enzyme by approximately 2-fold (Figure 2A, close circles; Table 1), while the m-NAD(P)-ME_R67A/R91A, m-NAD(P)-ME_K57S/E59N/K73E/D102S and c-NADP-ME enzymes cannot be activated by fumarate (Figure 2, open circles, closed triangles and open triangles, respectively; Table 1). Mesaconate is a *trans* dicarboxylic acid with 2-methyl group substitution (Figure 1). Although it can activate the enzyme by approximately 1.7-fold (Table 1), the concentration needed for half-maximal activation is substantially higher than that of fumarate (Figure 2B, closed circles).

**Table 1:** Effect of fumarate analogs on human m-NAD(P)-ME and c-NADP-ME^1^^,^ 2.

	3 ^,^ 4m-NAD(P)-ME WT	3 ^,^ 4m-NAD(P)-ME R67A/R91A	3 ^,^ 4m-NAD(P)-ME K57S/E59N/K73E/D102S	3 ^,^ 5c-NADP-ME WT
Fumarate	1.98±0.14	0.98±0.06	0.97±0.04	1.01±0.04
Mesaconate	1.75±0.16	1.01±0.04	0.99±0.05	1.02±0.03
*trans*-Aconitate	1.15±0.03	1.01±0.05	0.98±0.03	0.99±0.04
Monomethyl fumarate	2.78±0.08	1.07±0.03	1.17±0.05	0.99±0.03
Monoethyl fumarate	1.83±0.04	0.98±0.04	1.01±0.05	1.01±0.03
Oxaloacetate	1.00±0.03	0.94±0.02	0.87±0.02	1.02±0.03
Diethyl oxalacetate	0.31±0.03	0.85±0.02	0.91±0.03	0.93±0.02
Dimethyl maleate	1.00±0.02	0.97±0.02	0.95±0.03	0.99±0.03
Diethyl maleate	0.88±0.03	0.99±0.03	0.95±0.03	1.01±0.02
Dimethyl fumarate	0.79±0.03	0.99±0.02	0.98±0.02	0.99±0.02
Diethyl fumarate	0.65±0.04	0.92±0.01	0.94±0.02	1.01±0.03

1 These values were the ratios of specific activities of the enzyme determined in the presence and absence of these chemical compounds.

2 These values were the average with standard deviation of three-time repeats.

3 The enzyme specific activities of the m-NADP-(P)-ME WT, R67A/R91A, K57S/E59N/K73E/D102S and c-NADP-ME WT were approximately 0.1, 0.005, 0.02 and 0.08 µmol/min, respectively, and the final enzyme concentration in an individual assay was 20, 400, 100, and 25 ng/µl, respectively.

4 For m-NAD(P)-ME, the final concentrations of these analogs were fixed at 5 mM, except for monoethyl fumarate and mesaconate, which were fixed at 10 mM and 20 mM, respectively.

5 For c-NADP-ME, the final concentrations of these analogs were fixed at 2 mM.

*Trans*-aconitate is also a *trans* dicarboxylic acid but with a 2-carboxylate group addition (Figure 1). This compound showed a slight activation of m-NAD(P)-ME (Figure 2C, closed circles; Table 1). Single methyl or ethyl group substitutions of the terminal carboxylate of fumarate (Figure 1) had no significant influence on the enzyme activation of these two fumarate analogs. Monomethyl and monoethyl fumarate displayed activating effects similar to fumarate (Figure 2, D and E, respectively, closed circles; Table 1), indicating that the binding modes of these two mono-substituted fumarates were not significantly changed.

### Allosteric Inhibition of Human m-NAD(P)-ME by Fumarate and its Analogs

**Figure 3:**
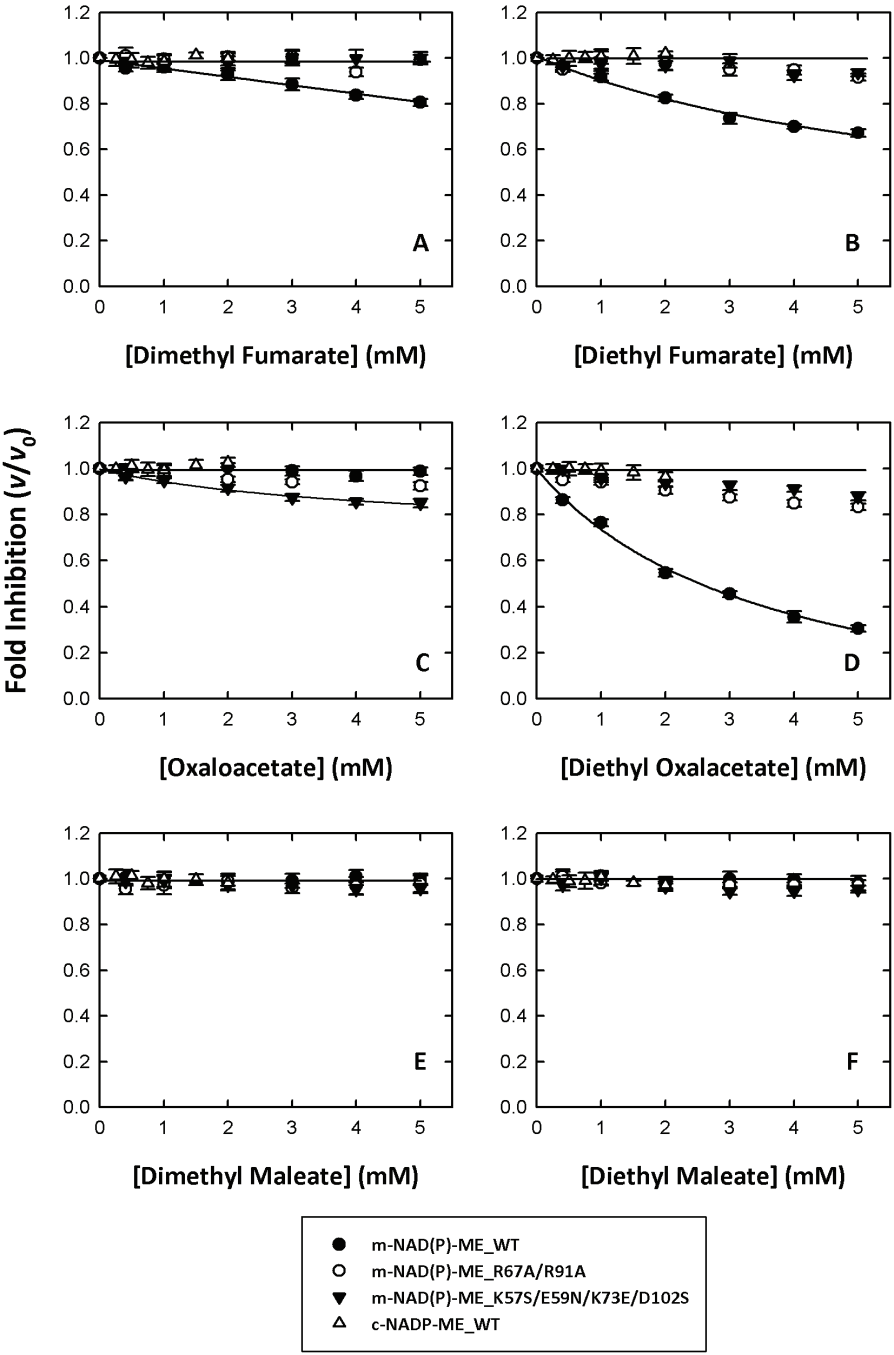
Allosteric inhibition of human m-NAD(P)-ME by fumarate and its analogs. (A) Dimethyl fumarate; (B) Diethyl fumarate; (C) Oxaloacetate; (D) Diethyl oxalacetate; (E) Dimethyl maleate; (F) Diethyl maleate. Closed circles, m-NAD(P)-ME; open circles, m-NAD(P)-ME_R67A/R91A; closed triangles, m-NAD(P)-ME_K57S/E59N/K73E/D102S; open triangles, c-NADP-ME. The specific activities of the m-NADP-(P)-ME WT, R67A/R91A, K57S/E59N/K73E/D102S and c-NADP-ME WT were approximately 0.1, 0.005, 0.02 and 0.08 µmol/min, respectively, and the final enzyme concentration in an individual assay was 20, 400, 100, and 25 ng/µl, respectively. The v and v_0_ represented the enzyme activity in the presence and absence of fumarate analogs, respectively.

Dimethyl or diethyl substitutions on both terminal carboxylates of fumarate (Figure 1) showed opposite effects on m-NAD(P)-ME. Dimethyl and diethyl fumarate inversely inhibited enzyme activity (Figure 3, A and B, respectively, closed circles; Table 1). Of the TCA cycle intermediates, fumarate and succinate are activators of the enzyme, but α-ketoglutarate (α-KG), the five-carbon α-ketodicarboxylic acid, is an inhibitor of ME.^29^ Oxaloacetate (OAA), a four-carbon α-ketodicarboxylic acid, had little effect on m-NAD(P)-ME enzyme activity (Figure 3C, closed circles; Table 1). However, once the ethyl groups were substituted on both terminal carboxyl groups of OAA (Figure 1), the diethyl oxalacetate showed significant inhibition on m-NAD(P)-ME enzyme activity (Figure 3D, closed circles; Table 1). The IC_50_ value of diethyl oxalacetate was approximately 2.5 mM.

Maleate, the *cis* isomer of fumarate, significantly inhibited ME activity [29]. However, dimethyl and diethyl maleate showed little inhibition of the enzyme activity (Figure 3, D and E, respectively, closed circles; Table 1). The fumarate analogs had activating or inhibiting effects on m-NAD(P)-ME (Figure 2 and 3, closed circles; Table 1) at different levels; however, they had no noticeable effects on c-NADP-ME (Figure 2 and 3, open triangles; Table 1). Furthermore, no further fumarate activation was displayed in the fumarate-binding abortive mutants, m-NAD(P)-ME_R67A/R91A and m-NAD(P)-ME_K57S/E59N/K73E/D102S (Figure 2 and 3, open circles and closed triangles, respectively; Table 1). Therefore, these fumarate analogs supposedly bind to the allosteric pocket of m-NAD(P)-ME at the dimer interface.

**Figure 4:**
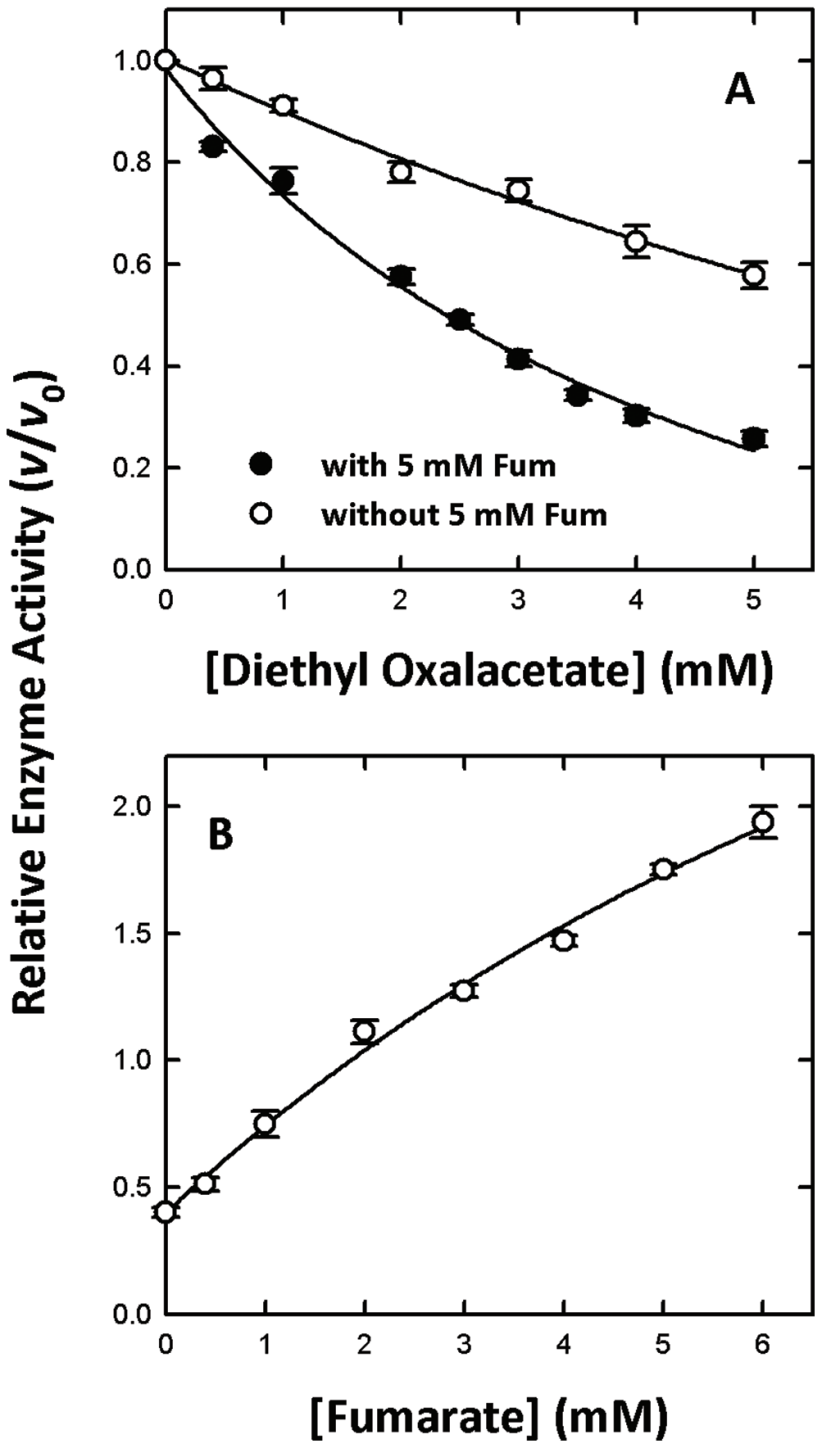
Effect of fumarate on diethyl oxalacetate-inhibited m-NAD(P)-ME activity. (A) The diethyl oxalacetate inhibition experiment of WT m-NAD(P)-ME without (closed circles) or with (open circles) 5 mM fumarate. (B) The fumarate rescue experiment of WT m-NAD(P)-ME. The enzyme was preincubated with 3 mM diethyl oxalacetate, and then the activity was restored with increasing concentrations of fumarate. The specific activities of the m-NADP-(P)-ME WT was approximately 0.1 µmol/min and the final enzyme concentration in an individual assay was 20 ng/µl. The v and v_0_ represented the enzyme activity in the presence and absence of fumarate analogs, respectively.

We found that diethyl oxalacetate may act as an allosteric inhibitor of the enzyme. To investigate this possibility, diethyl-oxalacetate inhibition experiments without or with fumarate were performed (Figure 4A, closed and open circles, respectively). It was clear that the inhibition of m-NAD(P)-ME by diethyl oxalacetate was decreased if fumarate was present (Figure 4A). Moreover, we examined the effect of fumarate on the rescue of the diethyl oxalacetate-inhibited m-NAD(P)-ME enzyme activity (Figure 4B). The m-NAD(P)-ME enzyme was first preincubated with 3 mM diethyl oxalacetate. The residual enzyme activity increased from 50% to over 100% with increasing fumarate concentrations, indicating that fumarate not only restores the enzyme activity but also activates the enzyme further. The above results also implied that diethyl-oxalacetate competes with fumarate in the allosteric pocket at the dimer interface.

## Conclusion

This paper reported an allosteric inhibitor of human m-NAD(P)-ME, diethyl oxalacetate. Because the binding of the allosteric inhibitors may impede the conformational change from open form to closed form, this paper may provide another rationale in designing allosteric inhibitors of the human m-NAD(P)-ME, a molecular target for cancer biology [11], [20].

## Materials and Methods

### Chemicals

L(-)-malate, fumarate, mesaconate, *trans*-aconitate, monomethyl fumarate, monoethyl fumarate, oxaloacetate, diethyl oxalacetate, dimethyl maleate, diethyl maleate, dimethyl fumarate and diethyl fumarate were purchased from Fluka (Buchs, Switzerland).

### Expression and Purification of the Recombinant m-NAD(P)-ME and c-NADP-ME

The protocols for the preparation of human m-NAD(P)-ME and c-NADP-ME have been previously described [30]–[32]. For m-NAD(P)-ME, the pRH281 expression plasmid was used to carry the gene, and expression was controlled by a *trp* promoter, which was modulated by the addition of *β*-indol-3-acetic acid (IAA). The expression vector was transformed into *Escherichia coli* BL21 cells to overexpress human m-NAD(P)-ME. The overexpressed enzyme was then purified by ATP affinity chromatography (Sigma). For c-NADP-ME, the pET21b expression plasmid was used to carry the gene, and expression was controlled by an inducible T7 promoter system, which was modulated by the addition of isopropyl β-D-1-thiogalactopyranoside (IPTG). The expression vector was transformed into *E. coli* BL21(DE3) cells to overexpress human c-NADP-ME. The overexpressed enzyme was then purified using a HIS-Select Nickel Affinity Gel column (Sigma). The lysate-Ni-NTA mixture was washed with buffer (10 mM imidazole, 500 mM sodium chloride, 2 mM β-mercaptoethanol, and 30 mM Tris-HCl, pH 7.4) to remove the discarded proteins, and c-NADP-ME was subsequently eluted with elution buffer (250 mM imidazole, 500 mM sodium chloride, 2 mM β-mercaptoethanol, and 30 mM Tris-HCl, pH 7.4). The purified enzymes were buffer-exchanged and concentrated in a 30 mM Tris buffer (pH 7.4) using an Amicon Ultra-15 centrifugal filter device (Millipore) with a molecular weight cut-off of 30 kDa. Enzyme purity was examined by SDS-PAGE, and protein concentration was determined using the Bradford method [33].

### Site-directed Mutagenesis

Single and double mutants were constructed using the QuikChange kit (Stratagene); the human m-NAD(P)-ME expression vector (pRH281) was used as a template for mutagenesis. The following PCR primers were used: 5′-CTTCAAGGACTTCTACCTCCC**TCT**ATAGAGACACAAGATATTC-3′ for K57S; 5′-CGATTTCATAGAAACTTG**GAA**AAAATGACTAGCCCTTTGG-3′ for K73E; 5′-GTTTTATAGAATACTGCAA**TCC**GACATTGAGAGTTTAATGCC-3′ for D102S; 5′-CTACCTCCCTCTATA**AAC**ACACAAGATATTCAAGCC-3′ for K57S/E59N; 5′-CACAAGATATTCAAGCCTTA**GCG**TTTCATAGAAACTTGAAG-3′ for R67A, and 5′-CTACATAATGGGAATACAAGAA**GCG**AATGAGAAATTGTTTTATAG-3′ for R91A. The PCR reaction was performed with the *Pfu* DNA polymerase and was incubated at 95°C for 30 sec, 55°C for 1 min and 68°C for 2 min per kb of plasmid length for 16–20 cycles. The templates were digested with the *Dpn*I restriction enzyme, and the resulting plasmid containing the desired mutation was transformed into *E. coli* XL-1 cells (Stratagene). All mutation sites were checked by sequencing.

### Enzyme Activity Assay

Human m-NAD(P)-ME and c-NADP-ME were assayed in reaction buffer containing 50 mM Tris-HCl (pH 7.4), 15 mM L-malate, 1 mM NAD^+^/NADP^+^ and 10 mM MgCl_2_ with various concentrations of fumarate or its analogs in a total volume of 1 ml. The absorbance at 340 nm was continuously monitored in a UV/VIS spectrophotometer Lambda 25 (Perkin Elmer, USA). An absorption coefficient of 6.22 cm^−1 ^mM^−1^ at 340 nm for NAD(P)H was used in the calculations. The diethyl oxalacetate inhibition experiment was assayed with 50 mM Tris-HCl (pH 7.4), 10 mM malate (pH 7.4), 10 mM MgCl_2_ and 1.0 mM NAD^+^ without or with 5 mM fumarate and a series of diethyl oxalacetate concentrations, ranging from 0 to 5 mM. The fumarate rescue experiment was assayed with 50 mM Tris-HCl (pH 7.4), 10 mM malate (pH 7.4), 10 mM MgCl_2_ and 1.0 mM NAD^+^ with 3 mM diethyl oxalacetate and a series of fumarate concentrations, ranging from 0 to 6 mM. All of the calculations were performed using the Sigma Plot 10.0 software program (Jandel, San Rafael, CA).
